# CT-guided percutaneous drainage of abdominopelvic collections: a pictorial essay

**DOI:** 10.1007/s11547-021-01406-z

**Published:** 2021-08-20

**Authors:** Massimo De Filippo, Sara Puglisi, Fabiano D’Amuri, Francesco Gentili, Ilaria Paladini, Gianpaolo Carrafiello, Umberto Maestroni, Paolo Del Rio, Francesco Ziglioli, Francesco Pagnini

**Affiliations:** 1 Section of Radiology, Department of Medicine and Surgery, Parma University Hospital, Parma, Italy; 2 Unit of Radiology, IRCCS Cà Granda, Ospedale Maggiore Policlinico, Milan, Italy; 3 Department of Urology, Parma University Hospital, Parma, Italy; 4 Unit of General Surgery, Critical Care and Pain Medicine Division, Department of Medicine and Surgery, Parma University Hospital, Parma, Italy

**Keywords:** CT guided, Percutaneous drainage, Abscess, Fluid collections, Pseudocyst, Interventional radiology

## Abstract

CT-guided percutaneous drainage is a safe and effective procedure that allows minimally invasive treatment of abdominopelvic abscesses and fluid collections. This technique has become an alternative for surgery with lower morbility and mortality rates. In this pictorial essay, we aim at providing an overview of the technical approaches, the main clinical indications and complications of CT-guided percutaneous drainage, in order to provide a practical guide for interventional radiologists, with a review of the recent literature. The focus will be the CT-guidance, preferred when the interposition of viscera, vascular and skeletal structures, counteracts the ultrasound guidance.

## Introduction

CT-guided percutaneous drainage is an interventional procedure performed by radiologist that allows minimally invasive treatment of fluid collections, potentially anywhere in the body, in particular in the deeper or more posterior parts which are difficulty reachable by the US-guided technique.

The CT guidance has several advantages: a better vision in obese patients, being less operator dependent and ensuring a more stable position of the patient on the CT table. Principal limitations are the non-real-time view, instead of US-guidance, and the radiation exposure.

It is a relatively noninvasive procedure, and it is considered safe and effective. Success of CT-guided percutaneous drainage is related to proper patient selection, preparation and adequate procedural planning. The experience and an adequate training of the operator are two important elements in the outcome of the procedure. Unfortunately, there is no specific definition by institution as the Society of Interventional Radiology (SIR) or the Accreditation Council for Graduate Medical Education (ACGME) regarding what constitutes an adequate training, as medical practice and technologies are constantly evolving. It follows that it is difficult to establish a minimum number of procedures to perform in order to acquire the right experience, even for the inter-individual variability, and because the competence should be determined on the achievement of educational milestones and not on the number of procedures performed. However, it is of fundamental importance for the radiologist who wants to deal with interventional radiology to start during the residency, to have a background on which to proceed with advanced training.

## Technique

### Pre-procedural assessment

The periprocedural management of patients undergoing imaging-guided procedures is a continually evolving paradigm. Moreover, local factors such as procedure type and patient conditions (renal, cardiac and liver function) may impact the final outcome. The Society of Interventional Radiology (SIR) has proposed general recommendations both for hematologic status and antibiotic prophylaxis of the patient. Intraabdominal and retroperitoneal abscess drainages are considered Moderate Risk Bleeding procedure; for this reason, pre-procedure laboratory coagulation tests are mandatory. INR above 1.5 should be corrected with orally or intravenously Vitamin K administration, or with fresh frozen plasma infusion and a platelets counts < 50.000/mcL should need prophylactic platelets transfusion; there is no consensus for the management of Activated PTT; however, a trend toward correcting for values > 1,5 times control is suggested in patient receiving intravenous infractionated heparin. Antiplatelet agent management consists in witholding Plavix 5 days before the procedure. In case of treatment with Novel Oral Anticoagulants, the procedure should be deferred until off medication. Aspirin or non-steroidal anti-inflammatory drugs do not alter routine coagulation testing, and it can be unacknowledged. During therapeutic low-molecular-weight heparin treatment, one dose before the procedure should be suspended. It is necessary to stratify the risk of the patient according to his coagulative status and the site of procedure: cardiovascular and thromboembolic risk must be weighted against the risk of bleeding. The patient recent medication administration records should be reviewed before the procedure taking into account different half-lives (Table 1) [1, 2].

**Table 1:** Summary of tests and the component of the coagulation function they assess, along with normal values [1]

Test	Indication	Normal range
INR/PT	Extrinsic pathway (I, II, V, VII, X) Oral anticoagulant therapy Liver disease	INR, 0.9–1.1
Activated PTT	Intrinsic pathway (VIII, IX, XI, XII) Intravenous heparin therapy von Willebrand disease Factor VIII, IX, or XI deficiency	Activated PTT, 25–35 s
Platelet count	Known or suspected thrombocytopenia	150,000–450,000/μL
Bleeding time	No current indication before imaging-guided procedures	

Antibiotic Prophylaxis in percutaneous drainage is usually done to prevent infection resulting from the communication created by a needle or a catheter. It is demonstrated that antimicrobial agents administration before inoculation show the best response. Several studies demonstrate that a single dose just before commencement of the procedure is as effective as a multiple-dose protocol.

Abscesses are typically polymicrobial: Gram-negative rods and anaerobes (E. Coli, Bacteroides fragilis, and Enterococcus) are commonly found in intrabdominal collection and Enterobacter species and anaerobes in pyogenic liver abscess. In asymptomatic patients is preferred to avoid unnecessary wide-spectrum coverage by awaiting the results of culture specimens: more current therapies include second- or third-generation cephalosporins (Cefoxitin 1 gr every 6 h, ceftriaxone 1gr every 24 h) or a combination of clindamycin and gentamicin in patients with a penicillin allergy, even though no consensus on the first choice antibiotic has been found [3]. Patients undergoing hepatic percutaneous abscess drainage should have prophylactic treatment with intravenous antibiotics, given the potential risk of biliary sepsis, even though asymptomatic [4, 5].

Patients with symptoms are often already being treated with antibiotics before undergoing to a percutaneous drainage. Empiric antibiotic coverage is recommended in those patients with clinical signs and symptoms (fever, leukocytosis) before the procedure, according to The SIR Practice Guideline.

Non-vascular-abdominal interventions can be performed under conscious or moderate sedation with intravenous administration of Fentanyl citrate and/or midazolam hydrochloride, showing high level of effectiveness and safety to avoid pre-procedural anxiety and procedural pain. Moderate sedation provides sufficient anxiolysis and control of unwanted movements during most radiologic interventional procedures; moreover, it reduce typical adverse reaction as hypovolemia. Although anesthesiologists are the best equipped to provide sedation and analgesia, they are usually not available: the provision of sedation and analgesia by properly trained non-anesthesiologists is thought to be safe, if the proper methods of drug administration and patient monitoring are adhered to. Percutaneous local anesthesia with Lidocaine (1–2%) is also mandatory [6, 7]. Pediatric patients mostly require a higher level of sedation than in adults, possibly also general anesthesia. Topical anesthetic creams are useful substitutes to anesthetic injections, being more tolerable.

### Procedural phase

At the beginning of the procedure, a diagnostic CT scan is performed to assess the position of the collection and its connections with the adjacent structures. The patient positioning and the insertion site are chosen using radiopaque markers on the skin in order to ensure the best route to reach the lesion. An ordinary antisepsis drug (Iodopovidone or Chlorhexidine) is generally sufficient to guarantee a safe procedure for the patient, then an injection of a local anesthetic (up to 10 ml of Lidocaine) is used at the skin site as well as along the tract.

The radiologist can choose between two different techniques to access the collection and to secure a drainage catheter. In both cases, a little (2–3 mm) skin incision is performed.

The Seldinger technique is a multi-step procedure: at first, the fluid collection is approached using a small, sharp hollow needle (18–22 Gauge Chiba needle). Once punctured, the stylet is withdrawn, and the fluid is aspirated through the trocar needle to confirm the correct intracavitary location. Care should be taken to avoid decompressing the collection completely prior to tube placement. A 0.038-in. floppy-tipped guidewire is advanced through the lumen of the trocar, and the needle is then withdrawn, leaving the distal tip of the wire coiled in the collection. Imaging at this point is useful to prove appropriate placement of the wire prior to track dilation. Fascial dilators are then advanced over the wire with a stepwise increase in diameter to dilate the intended track of the catheter. It may be helpful to mark the depth of the cavity on the side of the dilators to avoid excessive advancement and guide wire dislocation. It is mandatory to avoid kinking the guide wire while using the stiff dilators. Once the track is dilated, the drainage catheter (8–16 French, pigtail catheter) assembled with stiffener is advanced along the wire to reach the collection. After making sure that the catheter is positioned correctly into the collection, the stiffener is removed with the guiding wire and catheter is fixed to the skin.

On the other hand, the trocar technique is a single-step procedure consisting in a direct puncture with a standard trocar tip drainage catheter, composed of a stiffening cannula and sharp inner stylet in a catheter coaxial system.

After the access to the fluid collection, the catheter is moved forward, the stiffener and stylet are deflected and retained in place with a pigtail locking device.

The difference between the two techniques is that the trocar technique is more rapid than the Seldinger technique and can be performed by an operator without the aid of an assistant. It is the technique of choice for considerable or superficial fluid collections, and it is largely employed for endocavitary drain placement when continued dilation and guide wire placement is problematic.

The distal end of the catheter is linked to a drainage system through a three-way stopcock. Usually, the drainage system of choice is the closed one, since the fluid collections are mostly deep and the risk of infection is lower with this kind of drainage. After the procedure, the care of the catheter is vital. The closed drainage system allows proper irrigation of the catheter, which should be done preferably every 8 h with at least 10 mL of sterile saline solution. It is important to train the patient in the care of the drainage, since the dimission date is previous to its removal [8, 9]. Usually, the follow-up imaging is performed only in patients who are not improving clinically, and the removal of the drainage is based on the patient’s clinical and laboratory response.

## Clinical indications

### Abdominal fluid collections

**Fig. 1:**
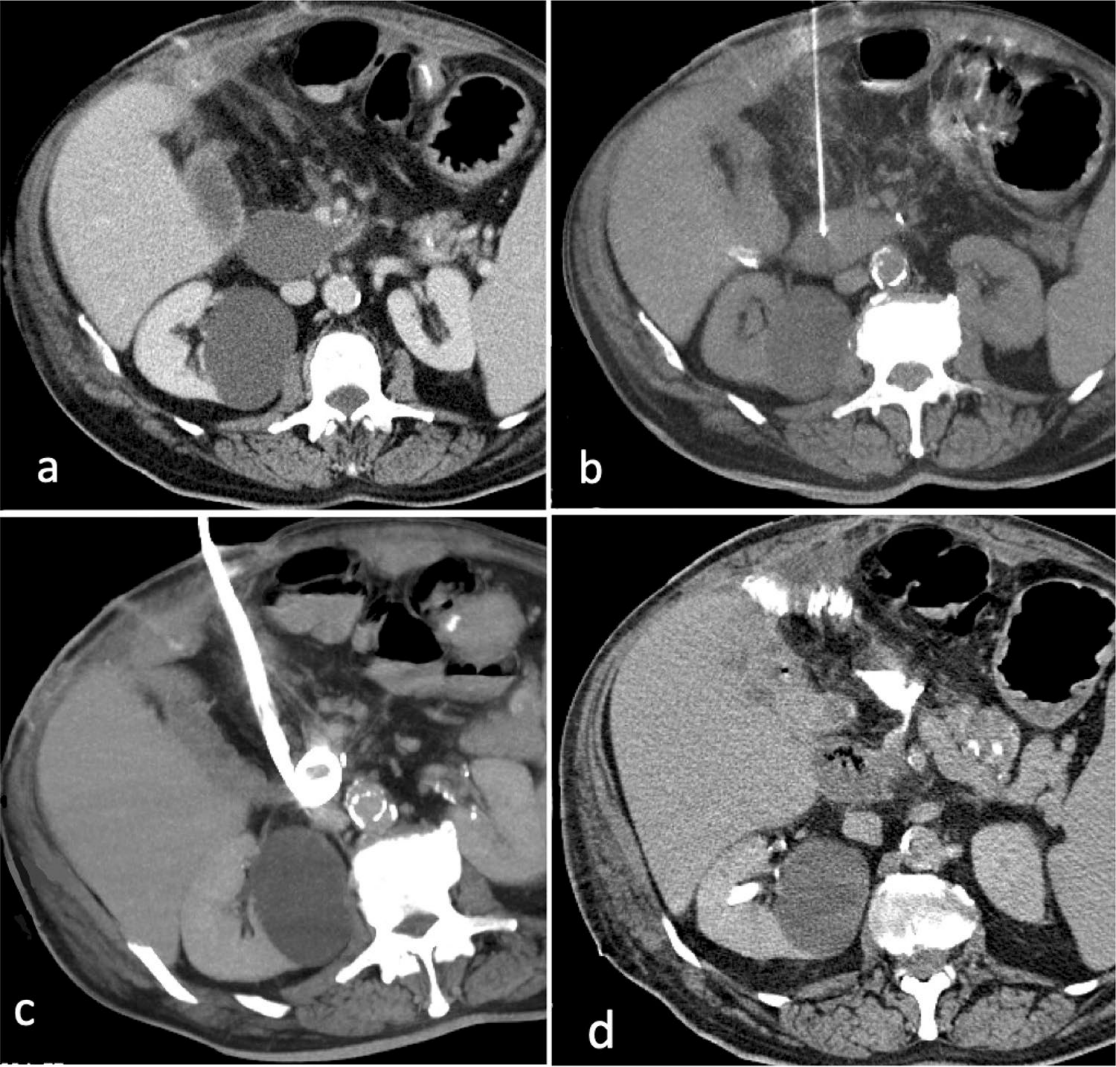
67-year-old male patient with a fluid collection in the inferior duodenal fossa, in proximity of the inferior vena cava, aorta and mesenteric vessels, after pancreatic surgery (**a**). After the puncture with a chiba needle (**b**), a 12F PIGTAIL catheter is placed (**c**: MIP reconstruction) using the Seldinger technique. Control CT scan after 2 weeks shows complete resolution of the fluid collection (**d**)

Abdominal collections can be subsequent to multiple inflammatory conditions including diverticulitis, appendicitis and Crohn’s disease but may also develop as a complication to recent intraabdominal surgery (Fig. 1) [10–12].

**Fig. 2:**
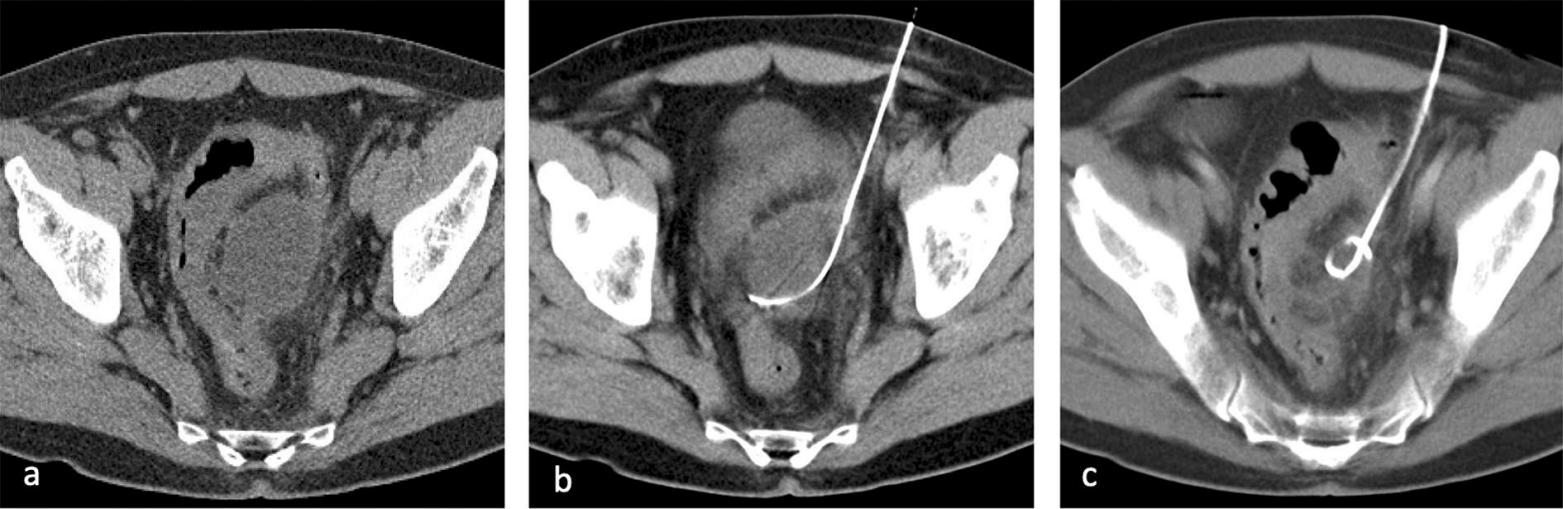
51-year-old patient with a perisigmoid diverticular abscess (**a**). After the procedural planning, an anterior approach is performed, placing the guide wire through a chiba needle (**b**) and subsequently a 14F PIGTAIL catheter (MIP-reconstruction, **c**)

If the fluid collection is smaller than 3 cm, it can be treated conservatively with antibiotics. Peridiverticular abscesses, which are the most recurrent complication of diverticulitis, can benefit of CT-guided drainage positioning because it allows improving clinical symptoms and temporizing for a later surgical approach, if necessary (Fig. 2) [13].

Appendicitis and Crohn’s disease can worsen with perforation, peritonitis or both, producing also periappendicular and perienteric abscesses and collections which can be efficiently treated with the positioning of a CT-guided drainage and the use of broad spectrum antibiotics [14].

### Hepatic abscesses and cysts

CT-guided percutaneous abdominal drainage (PAD) has become the leading choice for the treatment of multiple liver abscesses even in the presence of multiple septations and possible biliary connections [15, 16]. It is important to make sure to administer pre-procedural antibiotics, since the risk of transient septicemia after drainage can be accounted in 26% of cases [3]. Amoebic abscess is quite responsive to antibiotics (metronidazole) alone and requires drainage only in case of failure of medical management and diameter greater than 6–8 cm. Hydatid cysts must be treated due to frequent infection, biliary tree invasion and spread to other organs. Even though surgical approach is the gold standard treatment, percutaneous drainage is a valid alternative in the treatment and prevention of relapses.

### Splenic abscesses

CT-guided PAD offers an effective alternative to surgery in the therapeutic management of splenic abscesses. Optimal access to a splenic abscess should traverse the least amount of normal splenic parenchyma as possible.

Complications of splenic PAD include sterile or infected pleural collections derived from pleural crossing during catheter placement which can be limited by careful pre-procedural planning under CT [17]. As the access point for hepatic and splenic abscesses is often high and may cross the pleura, post-catheter placement imaging should screen for a pneumothorax. Hemorrhage is a well-described post-procedural complication of splenic drainage, and it is recommended that patients are monitored closely for this complication [18, 19].

### Pancreatic collections, pseudocysts and abscesses

**Fig. 3:**
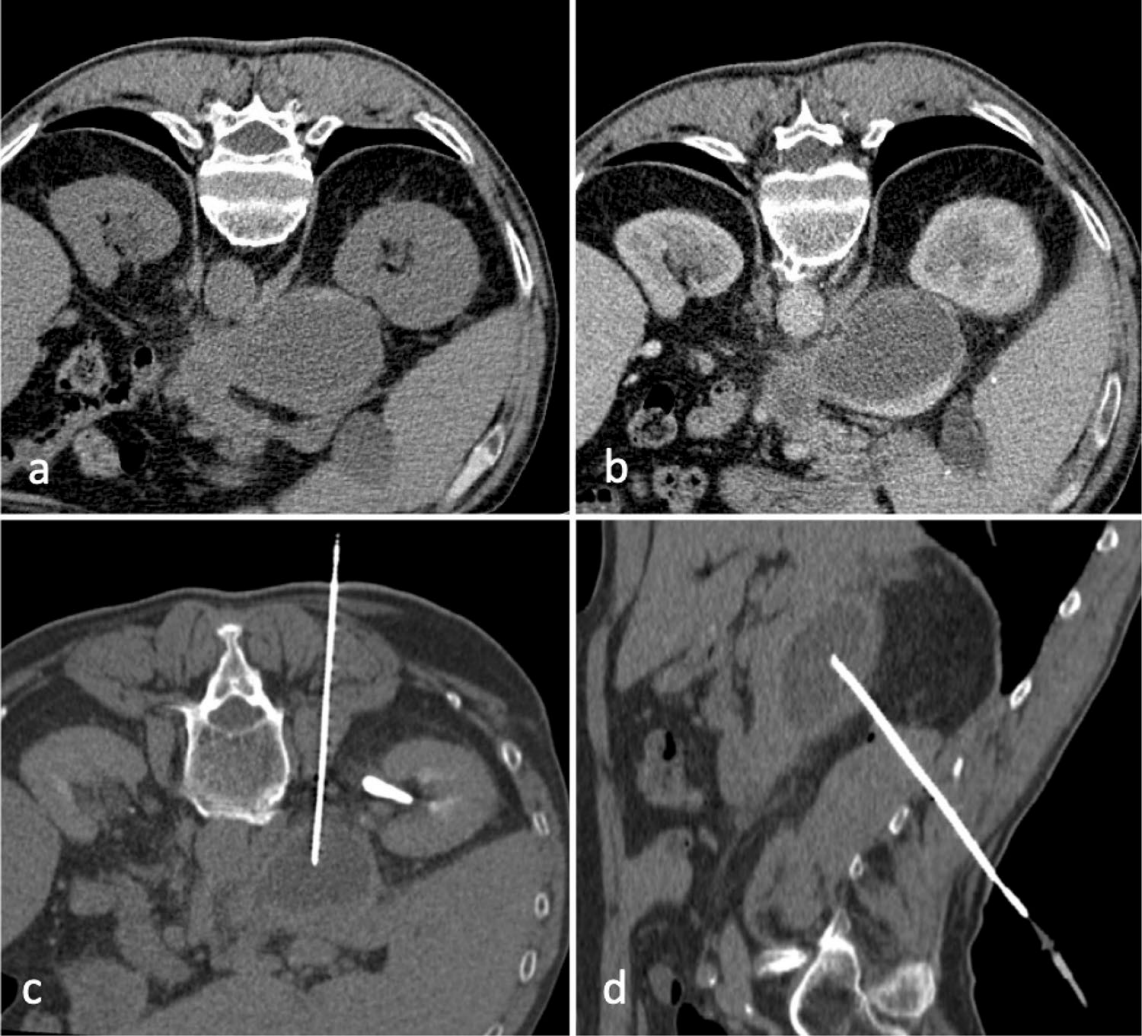
Drainage of a pancreatic pseudocyst in a 65-year-old male patient. Axial pre- and post-contrast CT scans (**a**, **b**) point out the interposition of the lung. Using MPR reconstructions, the lesion is reached through a caudocranial path and drained without complications (**c**, **d**)

Almost 40% of patients with acute pancreatitis develop peripancreatic fluid collections [20]. If acute pancreatitis-associated fluid collections are small, they do not require drainage; on the contrary, infected collections should be drained. Pancreatic pseudocysts which develop after 3–4 weeks from the acute event may require drainage when large or symptomatic (Fig. 3) [21].

CT-guided percutaneous drainage is the technique of choice for this kind of collection, because it provides the best visualization of the lesions and the correlation with the surrounding structures, particularly of vessels. The access can be made through the right pararenal space or through the gastrocolic ligament if the peripancreatic fluid collections are located near the pancreatic head; if they are in the premises of the tail, then the best access is through the left pararenal space. Peripancreatic collections should be managed with larger bore drain placement and require the positioning of multiple catheters.

The use of CT-guided percutaneous drainage in acute necrotic collections and walled-off necrosis is generally limited to stabilizing a critically ill patient prior to surgical debridement. Pancreatic and peripancreatic necrosis are generally not amenable to early drainage, but when the contents become liquid, CT-guided percutaneous drainage can be an option.

### Pelvic collections

**Fig. 4:**
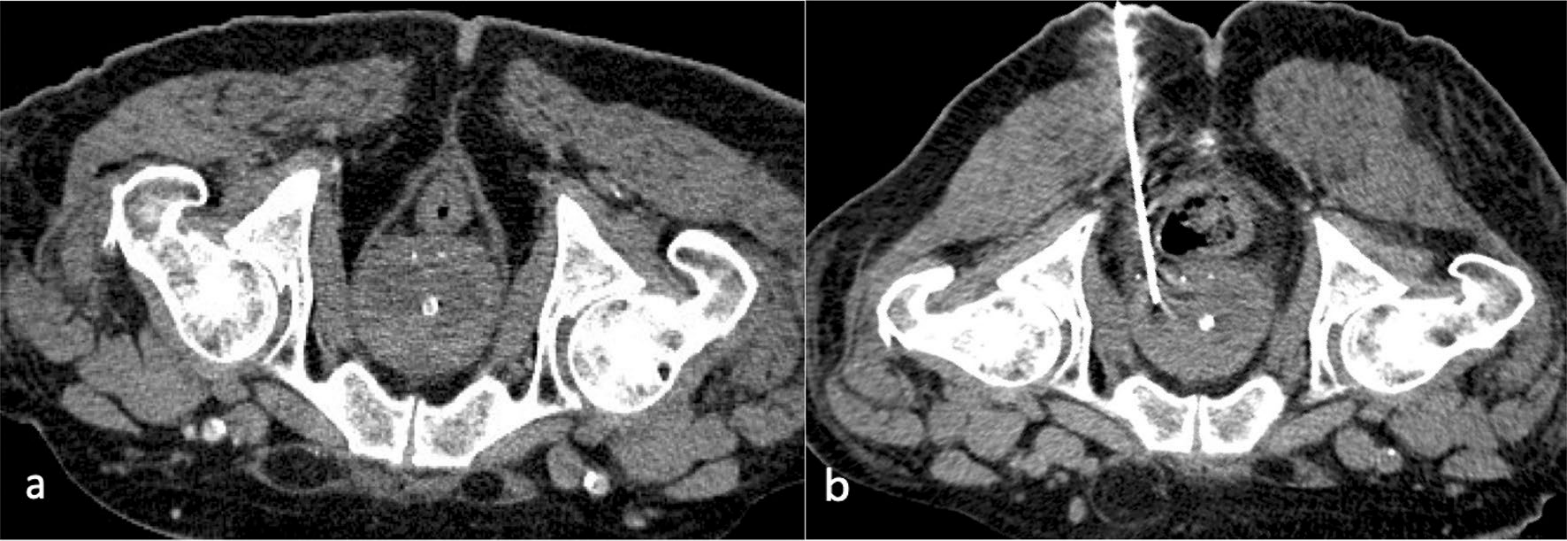
60-year-old male with pollakiuria, dysuria and urinary retention. CECT shows a prostatic abscess (**a**). The drainage is successfully performed through a left transgluteal approach (**b**)

Deep pelvic abscesses may be difficult to access because of the number of structures encountered during the path, both from an anterior and from lateral approach [22]. CT-guided percutaneous drainage can be performed using a transgluteal approach (Fig. 4).

**Fig. 5:**
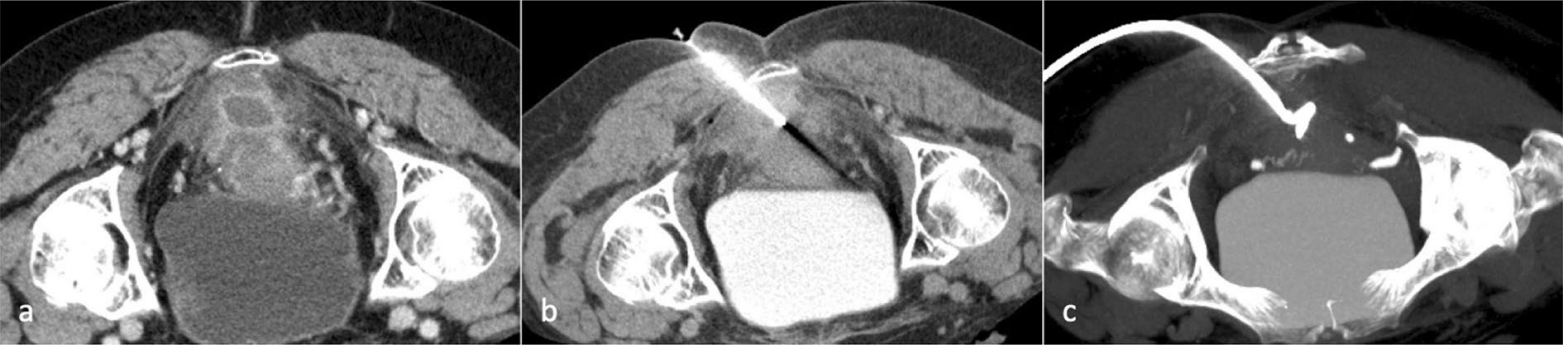
Pre-procedural enhanced CT scan shows a presacral abscess in a 65-year-old patient with Crohn’s disease (**a**). Through a transgluteal approach, a 12F PIGTAIL catheter is placed without complications (**b**, **c**)

The patient is placed in a prone or lateral position, and access is obtained through the gluteal muscles. The optimal route for this technique is near the sacrum at the level of the sacrospinous ligament, preferably below the piriformis muscle to avoid the sciatic nerve and vessels (Fig. 5).

The transgluteal approach is extremely painful for the patient although most of them can tolerate this procedure with appropriate analgesia [23]. An important complication is the formation of a kink in the guide wire during the advancement of the fascial dilators through the gluteal muscle; the employment of a stiffer guide wire seems to decrease this risk. Given the posterior location of the drain, care should be taken to drape the catheter tubing and stop-cock anteriorly for better patient comfort and easier catheter care.

In the setting of post-surgical lymphadenectomy in oncological diseases, it is frequent the development of lymphoceles, which can be drained from an anterior or posterior approach [24].

Moreover cystic formations of gynecologic pertinence may be likely to be percutaneously drained in patients unfit for surgery.

### Subphrenic collections

**Fig. 6:**
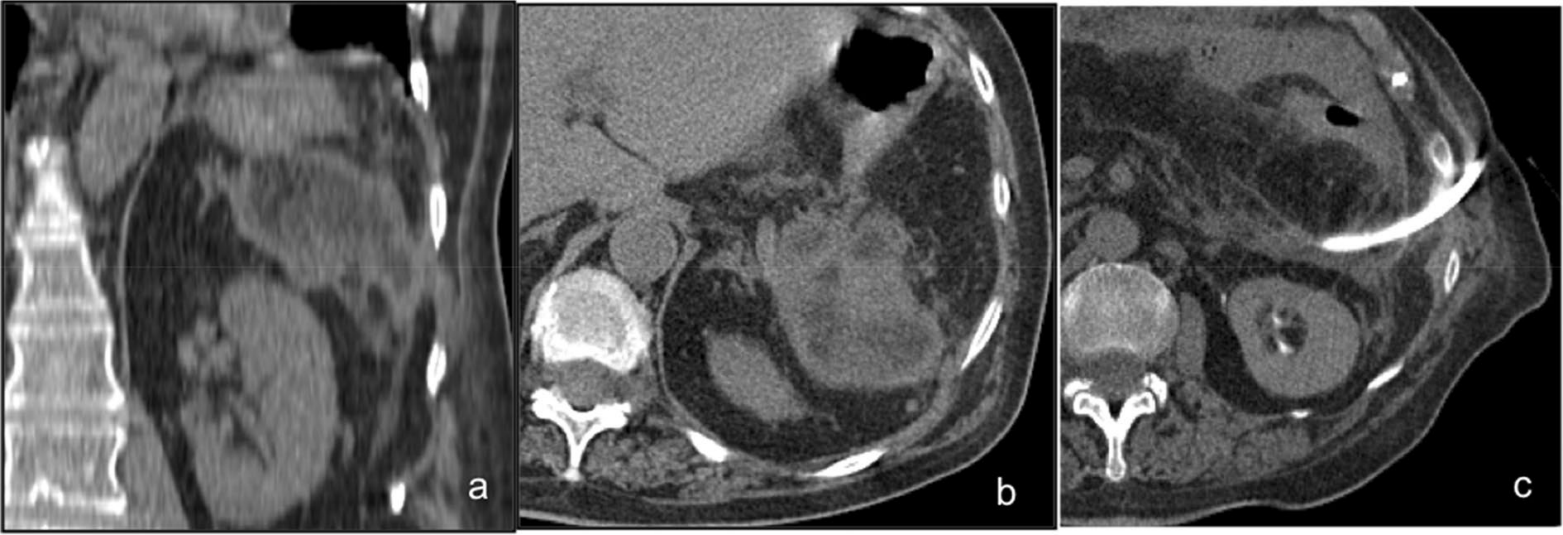
Coronal (**a**) and axial (**b**) CT scans of a 72-year-old patient with left subphrenic abscess after spleno-pancreatico-duodenectomy, drained with a 12F PIGTAIL catheter (**c**)

CT-guided percutaneous drainage is the treatment of choice for subphrenic fluid collections [25]. Given the proximity to the lung and pleural space, it is important for the radiologist to have a complete view of these structures when evaluating the possible access route [19]. A subcostal anterior approach provides a lower risk of passing through the pleura, but it may require angled gantry (Fig. 6).

Intercostal access should be performed with placement of the needle just above the rib to avoid the neurovascular bundle which passes caudally [26]. Intercostal approach is burdened with minor but frequent pleural complications, such as pleural effusions. CT-guided percutaneous drainage positioning can also bring to other complications such as empyema (rare) and pneumothorax, which can be found in a higher percentage of patients; for this reason, patients must be screened after procedure with a CT scan. If present, large and symptomatic pneumothorax should be treated with the positioning of a chest tube [9].

### Psoas collections 

**Fig. 7:**
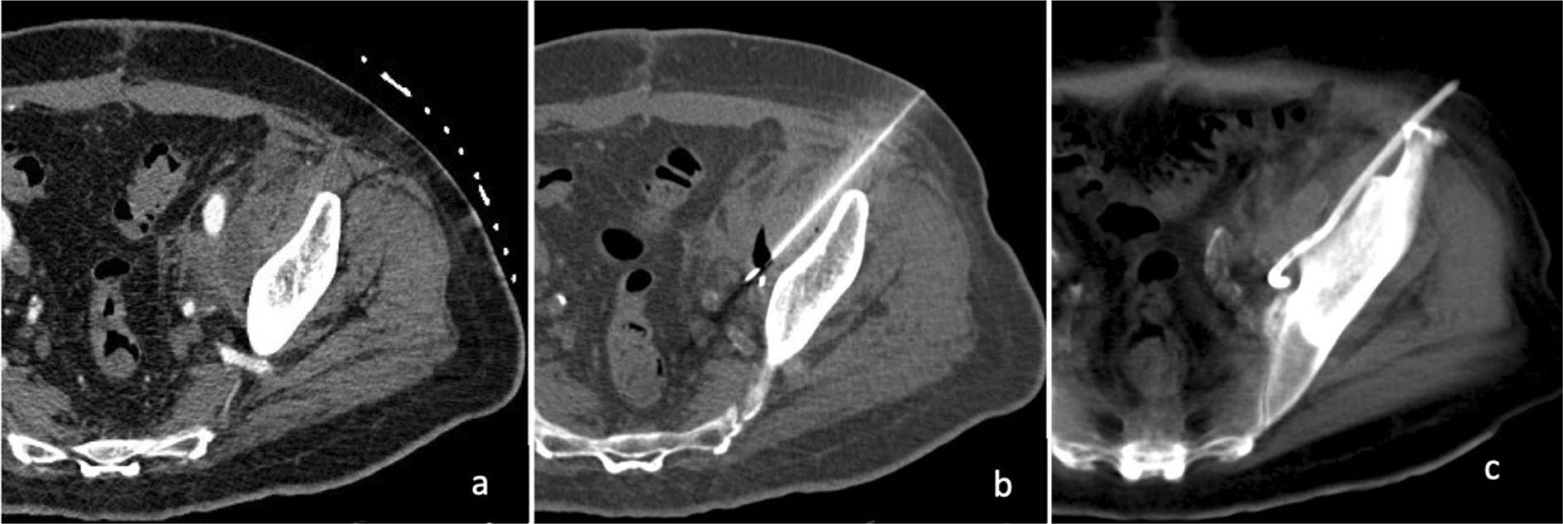
Left psoas abscess in a 76-year-old patient (**a**). The positioning of the guide wire (**b**) and the placement of a 12F PIGTAIL catheter (**c**) are carefully obtained under CT-guidance avoiding peritoneal cavity

Psoas and other retroperitoneal collections may be difficult to percutaneously drain because of their location. Proper planning, using CT, is vital to evaluate the presence of interposed structures on the path, like blood vessels or bowel (Fig. 7).

Iliopsoas collections can be primary or secondary, due to underlying diseases, which can be gastrointestinal or urinary. Only larger collections are amenable to CT-guided percutaneous drainage, and surgical intervention is not usually applied. Smaller psoas abscesses (inferior to 3 cm) are usually managed with medical treatment only [3, 20].

### Renal and perirenal collections

**Fig. 8:**
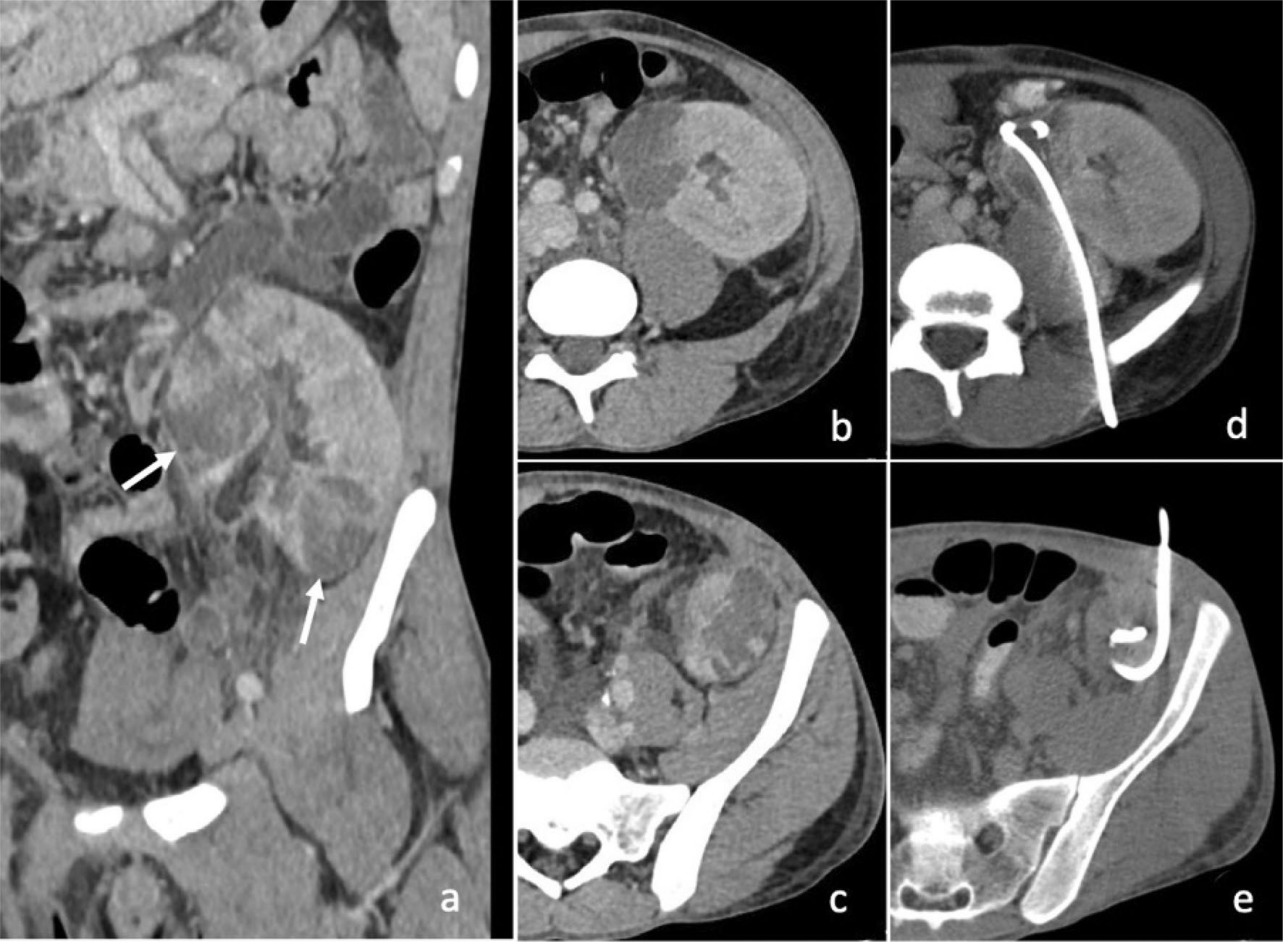
Coronal (**a**) and axial (**b**, **c**) enhanced CT scans of a 34-year-old patient with two abscesses in a transplanted kidney in the left iliac fossa (white arrows in **a**), drained with two 12F PIGTAIL catheters. The abscess of the upper pole of the kidney (**b**) required a posterior approach because of the interposed colon (**d**). The lower pole of the kidney was drained using an anterior approach (**c**, **e**)

Renal abscesses usually occur in patients presenting underlying predisposing conditions such as nephrolithiasis, pyonephrosis, hydronephrosis and transplanted kidney (Fig. 8) [27].

Perirenal collections can be due to surgery for cancer [28]. Drainage of these lesions is required when large, over 5 cm, whereas when they are smaller, can be managed by medical treatment alone. The reasons are basically due to the complications that large abscesses may cause: rupture in the perirenal space, but also in the vascular structures or in the collecting system [29].

### Pediatric patients

At pediatric age, image-guided drainage is a valid tool in treating collections secondary to inflammatory bowel disease, postoperative complications and acute appendicitis, in particular in the case of abscess formation [30]. In order to avoid radiation exposure, ultrasound imaging should be used when possible. If it is technically not manageable, then CT-guided percutaneous drainage should be implied optimizing CT parameters and shielding the most sensitive parts, like gonads or thyroid [31]. The approach to children is different than that to adults, especially regarding the care given before and after the procedure, which should be tailored to pediatric needs. Also, monitoring and resuscitation equipment have to be made to measure, heat loss must be minimized and an anesthesiologist should be present during the procedure, which is usually performed on sedation [32]. Once planning images are obtained, greater image noise is often acceptable for catheter check images. Post-procedure imaging with CT should be performed sparingly or avoided if possible, by relying more on clinical response, catheter outputs and ultrasound for decision making [33].

## Complications

CT-guided percutaneous drainage positioning may have some complications, such as fistula formation, bleeding and sepsis. Also, if the tube is not well-positioned, it can bring to inadequate drainage [34]. Some discomfort or scar formation at the site of insertion may be unavoidable. Involvement of the interventional radiologist in patient rounds is important for patient care, early detection of complications, exchange of information with referring physicians and continued learning. Although every effort is made to minimize the length of treatment, every patient is different, and catheter removal can sometimes take a long time.

There are many reasons why a collection may respond slowly to treatment, leading to prolonged drainage [35]. It is important to stress from the outset what can be realistically expected. The presence of loculations, the development of a fistula and the presence of a tumor mass can hamper catheter removal. Loculations can often be effectively treated by increasing the size of the drain or by repositioning it. Fistula development often results in long-term catheter placement. Diversion of upstream fluids (bile, urine, pancreatic juices, bowel content) by surgical or endoscopic means should be considered in complicated cases. CT-guided drainage of an infected tumor should be approached with extreme caution and should involve multidisciplinary discussion [36]. Catheters placed into an infected tumor can rarely be removed and often remain for life, or until surgical removal of the tumor.

Another frequent occurrence is catheter dislodgement: it is important to adequately secure drainage catheters and educate patients regarding the risk of pulling on to it. The decision to reinsert a drainage catheter depends on the reason for insertion, the adequacy of treatment and need for further drainage. Bleeding is not uncommon after catheter placement: it may be due to the presence of altered blood in a postoperative collection and is often self-limiting. However, occasionally, it can be a sign of significant injury such as pseudoaneurysm formation. In cases of concern, it is advisable to remove a catheter over a wire, so that the catheter can be replaced to tamponade significant bleeding as a temporary measure before definitive treatment by surgery or embolization.

## Conclusions

The method of choice for the imaging guidance is the ultrasound; however, collections of small dimensions and located in deep recesses require CT guidance.

The CT-guided percutaneous drainage positioning is a safe, effective and minimally invasive procedure which often allows to avoid the surgical treatment. The procedure plays a pivotal role in the management of patients with abdominal and retroperitoneal abscesses, and recovery period is usually shorter than after a laparotomic or laparoscopic surgical treatment.

Given the precise anatomical knowledge of the general radiologist, the CT-guided percutaneous drainage can be considered, with an appropriate training, an easily performed procedure instead of an exclusive prerogative of the interventional radiologist.
